# Engineered Polymeric Hydrogels for 3D Tissue Models

**DOI:** 10.3390/polym8010023

**Published:** 2016-01-20

**Authors:** Sujin Park, Kyung Min Park

**Affiliations:** Division of Bioengineering, College of Life Sciences and Bioengineering, Incheon National University, 119 Academy-ro, Yeonsu-gu, Incheon 22012, Korea; sujin.park@inu.ac.kr

**Keywords:** polymeric hydrogels, engineered tissue models, artificial extracellular matrices, tissue engineering, drug screening, basic cell biology

## Abstract

Polymeric biomaterials are widely used in a wide range of biomedical applications due to their unique properties, such as biocompatibility, multi-tunability and easy fabrication. Specifically, polymeric hydrogel materials are extensively utilized as therapeutic implants and therapeutic vehicles for tissue regeneration and drug delivery systems. Recently, hydrogels have been developed as artificial cellular microenvironments because of the structural and physiological similarity to native extracellular matrices. With recent advances in hydrogel materials, many researchers are creating three-dimensional tissue models using engineered hydrogels and various cell sources, which is a promising platform for tissue regeneration, drug discovery, alternatives to animal models and the study of basic cell biology. In this review, we discuss how polymeric hydrogels are used to create engineered tissue constructs. Specifically, we focus on emerging technologies to generate advanced tissue models that precisely recapitulate complex native tissues *in vivo*.

## 1. Introduction

Polymeric biomaterials are extensively used in the biomedical research fields due to their multi-tunable properties and easy fabrication [1,2]. Their physico-chemical and biological properties can be easily modulated by varying monomer components and incorporating bioactive molecules (e.g., proteolytic degradable sites, growth factor-binding moieties and cell adhesive sites) [3]. Polymeric biomaterials can be created by various fabrication methods, including salt-leaching, electrospinning, electrospraying, solvent casting and hydrogel formation [4]. Specifically, polymeric hydrogels, which present hydrophilic three-dimensional (3D) networks immersing a large amount of water, have been used in a wide range of biomedical applications, including tissue engineering, regenerative medicine, biosensors and in drug delivery systems [5,6,7,8,9,10].

Extracellular matrices (ECMs) are composed of structural proteins, polysaccharides and various soluble factors, which present a milieu of physical properties (e.g., pH, oxygen tension, mechanical properties and topology). It is well known that the cellular microenvironments play a critical role in cell growth, migration and differentiation into the native tissues [11]. Recently, polymeric hydrogel matrices have attracted attention as 3D artificial extracellular microenvironments due to their structural similarity to the native ECMs, which provide complex and convoluted cellular environments [12]. Using artificial ECMs, many researchers have developed engineered 3D tissue constructs for tissue engineering and regenerative medicine. Moreover, these engineered tissue models have been implicated as alternatives to animal models and traditional two-dimensional (2D) culture models for toxicity tests, the evaluation of drug efficacy and screening and for a better understanding of basic cell biology in healthy and pathological tissues. Many studies demonstrated that animal models and 2D tissue models showed critical limitations, such as interspecies-dependent discrepancies, an inability for real-time observation and a lack of dynamic experimental control [13].

Various types of polymeric hydrogels have been developed as 3D cellular microenvironments to create engineered tissue constructs. In this review, we discuss how polymeric hydrogels are currently used to create engineered 3D tissue models. Moreover, we introduce emerging technologies to generate advanced tissue models that accurately recapitulate *in vivo* cellular microenvironments, which are integrated with emerging tools, such as nano-/micro-fabrication techniques.

## 2. Polymeric Hydrogel Matrices

Polymeric hydrogels are 3D hydrophilic networks that can withstand large quantities of water [14]. Various approaches are utilized to create these 3D matrices, including physical and chemical crosslinking strategies [15,16]. Physical gels involve non-covalently crosslinked networks formed via crystallization and molecular entanglements and respond to changes in physical conditions (e.g., pH, temperature and shear stress) [17,18,19]. While these hydrogels have some benefits, such as reversibility and the absence of chemical reactions, they have disadvantages, including lower stability and mechanical properties compared to chemically-crosslinked hydrogels. Thus, they can be easily collapsed by various physical and chemical stresses in physiological conditions (e.g., ionic strength, pH and temperature). Chemically-crosslinked gels are formed by covalent bonds through Michael-type addition, click chemistry, Schiff-base crosslinking, disulfide crosslinking, photo-crosslinking and enzyme-mediated crosslinking reactions [20]. Chemical crosslinking strategies have some advantages, such as stable network formation either *in vitro* or *in vivo* with tunable mechanical properties. However, they have potential problems with biocompatibility and safety issues due to the chemical reagents and their *in situ* chemical reactions during hydrogel formation. As chemical crosslinking may be toxic to cells or even at the systemic level, many researchers have focused on developing biocompatible crosslinking approaches to create polymeric hydrogels for a wide range of biomedical applications.

**Figure 1:**
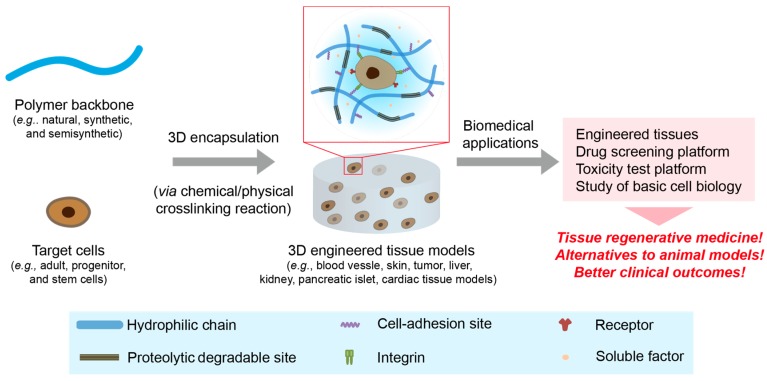
A schematic representation of engineered 3D tissue models for a wide range of biomedical research fields. The engineered 3D tissue models are created by encapsulating target cells via either chemical or physical crosslinking reactions. The engineered hydrogel matrices should be decorated or incorporated with proteolytic degradable sites, cell-adhesion moieties and growth factor-binding molecules to support cell growth within the matrices. Advanced tissue models have been utilized as a platform in a broad range of biomedical research fields, including the study of basic cell biology, tissue regeneration and drug screening/toxicity test platforms.

Recently, polymeric hydrogels have emerged as 3D cellular microenvironments to recapitulate aspects of the native ECMs, which have various physico-chemical properties, including gradients of nutrients, pH, oxygen, stiffness and topography [21,22]. To create these biomimetic microenvironments, the matrices should be decorated with cell-adhesion sites (e.g., Arg-Gly-Asp, RGD), proteolytic degradable sites (e.g., matrix metalloproteinase (MMP)-sensitive sites) and growth factor-binding sites (e.g., heparin or its derivatives), which are critical for supporting 3D cell growth and regulating their fate [16,23,24]. Their physico-chemical properties, including stiffness, swelling ratio, porosity and nutrient/gas permeability, can be controlled easily by varying the crosslinking density in the 3D networks, which is a pivotal advantage of polymeric hydrogels as an artificial cellular microenvironment. These multi-tunable hydrogel matrices provide dynamic and convoluted 3D artificial extracellular microenvironments to better mimic the native ECMs. With advances in polymeric hydrogel materials, various kinds of hydrogels have been developed as 3D-engineered matrices to generate 3D-engineered tissues as an emerging platform for the study of basic cell biology, tissue regenerative medicine, drug discovery and toxicity test (Figure 1). In this section, we discuss the fabrication of representative hydrogel materials using natural, synthetic and semi-synthetic polymers.

### 2.1. Natural Hydrogels

Natural polymers (e.g., collagen, gelatin, fibrin, hyaluronic acid (HA), dextran, alginate, cellulose and heparin) are promising polymeric biomaterials to create artificial ECMs due to their inherent biocompatibility, bioactivity and biodegradability. Among these bioactive molecules, collagen has been extensively used as a 3D matrix to provide cellular microenvironments due to its cellular responsibility, such as cell adhesive and proteolytic degradable sites, which are crucial for regulating cell to matrix interactions and ECM remodeling [25]. Collagen hydrogels are fabricated by thermogelation and chemical crosslinking. The cells or tissues can be easily encapsulated within the 3D matrices during hydrogel formation. These unique properties make collagen a promising material for generating 3D artificial cellular microenvironments. While natural polymers have many benefits as engineered 3D matrices, there are some limitations, such as weak mechanical properties, the potential for disease transmission, possible immunogenic reactions, fast degradation and batch-to-batch variability [12]. Therefore, researchers are developing synthetic hydrogel materials to overcome these limitations.

### 2.2. Synthetic Hydrogels

Various synthetic hydrogels are engineered to generate 3D cellular microenvironments, including poly(ethylene glycol) (PEG), poly(vinyl alcohol) (PVA), poly(*N*-isopropylacrylamide) (PNIPAAm) and poly(ethylene oxide)–poly(propylene oxide)–poly(ethylene oxide) (PEO–PPO–PEO) [14]. Synthetic hydrogels possess an exact composition, multi-tunable properties and biocompatibility. Among them, PEG-based hydrogels are the representative matrices that recapitulate the native ECMs due to their biocompatibility, high swelling properties and multi-tunable properties. However, PEG hydrogels should be tailored with cellular active sites to support cell growth within the 3D matrices due to bioinert properties [12]. The PEG-based hydrogels are decorated with cell adhesion molecules and MMP-sensitive peptide sequences or contain bioactive natural polymers (e.g., collagen, gelatin, fibrin, HA) to enhance cellular activities and matrix remodeling for 3D cell culture. While these synthetic matrices have been widely utilized to create 3D tissue models, well-defined engineered matrices are still required to accurately mimic native cellular environments.

### 2.3. Semi-Synthetic Hydrogels

Growing evidence demonstrates that synthetic hydrogel materials have some limitations to precisely mimic the native ECMs. Thus, many researches are currently focusing on designing advanced hydrogel materials composed of chemically-modified natural polymers, defined as semi-synthetic hydrogels [26,27]. These hydrogel matrices provide precisely-controlled microenvironments with the bioactive features of natural materials and multi-tunable properties by varying the chemical parameters [8]. Among the various candidates, HA is a promising polymer backbone to engineer 3D hydrogel matrices. HA, a major component of natural ECMs, is an anionic non-sulfated glycosaminoglycan (GAG) consisting of d-glucuronic acid and d-*N*-acetylglucosamine [28]. It is well known that HA is a biocompatible, non-immunogenic, non-inflammatory and biodegradable natural polymer [5] with a binding affinity to cell surface receptors that regulate cellular behaviors, such as cell adhesion, proliferation, migration and differentiation [29,30]. Therefore, HA-based semi-synthetic hydrogels are utilized as 3D cellular microenvironments, which can be formed via various physical and chemical crosslinking reactions, including Michael-type addition, click reaction, enzyme-mediated crosslinking reactions, shear stress and host guest reactions [31]. With these approaches, many researchers have developed engineered cellular microenvironments to precisely recapitulate the spatiotemporal complexity of native tissues.

## 3. Engineered 3D Tissue Models

With advances in biomaterials engineering, various engineered tissues have been developed for biomedical applications. Specifically, these artificial tissue constructs are a promising platform for tissue transplantation, a better understanding of basic cellular biology in healthy and diseases and as an alternative to animal models for drug screening and toxicity tests (Table 1). In this section, we discuss various engineered 3D tissue models created by the emerging fabrication techniques.

**Table 1:** Polymeric hydrogel matrices for engineered tissue models and their applications.

Type of polymer (polymer backbone)	Crosslinking method	Cell source	Engineered 3D tissue models	Applications	Reference
Natural (collagen)	Thermogelation	NHEKs, NHDFS, SCC-12B and SCC13	Skin tissues (*in vitro* models for normal skin and human cutaneous SCC)	- Studying the molecular mechanism of carcinoma progression; - Assess the effect of EGFR activation and inhibition on SCC progression	[32]
Natural (collagen)	Thermogelation	ADSCs	Skin tissues (tissue-engineered dermo-epidermal skin grafts)	- Evaluating prevascularized skin graft	[33]
Synthetic (PEG)	Chemical crosslinking (click-chemistry)	ECs and mural cells (MSCS, SMCs, HDFs)	*In vitro* angiogenesis models	- Studying the regulation of heterocellular communication	[34]
Semi-synthetic (gelatin)	Chemical crosslinking (laccase-mediated crosslinking reaction)	ECFCs	Vascular tissues	- Creating 3D vasculatures; - Studying basic cell biology for the hypoxia effect on vascular morphogenesis	[22]
Semi-synthetic (HA/gelatin)	Chemical crosslinking (photo-crosslinking reaction)	GBM	Tumor models (brain tumor models)	- Studying the effect of spatial gradation on brain tumor cells	[35]
Semi-synthetic (HA)	Chemical crosslinking (Michael-type addition reaction)	HT1080 and ECFCs	Tumor models (tumor angiogenesis models)	- Investigating the effect of matrix stiffness and oxygen tension on vascular cell invasion	[36]
Semi-synthetic (HA)	Chemical crosslinking (click-reaction)	MCF-7, T-47D, SK-MEL-28 and MDA-MB-231	Tumor models (tumor invasion models)	- Studying the effect of matric stiffness and cell adhesion ligand density on cancer cell invasion	[31]
Semi-synthetic (PEG/heparin)	Chemical crosslinking (maleimide-mediated crosslinking reaction)	HUVECs, MSCs, MCF-7, MDA-MB-231, LNCaP, PC3	Tumor models (tumor angiogenesis models)	- Tri-culture systems to investigate the effect of cell components on tumor angiogenesis and drug resistance	[37]
Semi-synthetic (PEG)	Chemical crosslinking (photo-crosslinking reaction)	Hepatocytes	Liver models (hepatic tissue models)	- Investigating the effect of hepatocyte density on the *in vitro* function of hepatic tissues; - Liver tissue regeneration	[38]
Semi-synthetic (PEG)	Chemical crosslinking (photo-crosslinking reaction)	Human embryonic stem cell-derived pancreatic precursor cell aggregates	Pancreatic islet models	- Studying the effect of collage type I on islet aggregate formation and their viability within the microenvironment	[39]

ADSCs, adipose-derived stem cells; ECs, endothelial cells; ECFCs, endothelial colony-forming cells; EGFR, epidermal growth factor receptor; GBM, glioblastoma multiforme; HA, hyaluronic acid; HDFs, human dermal fibroblasts; HT1080, human fibrosarcomas; MCF-7: human breast adenocarcinoma cell line; MDA-MB-231, human breast adenocarcinoma cell line; MSCs, mesenchymal stem cells; NHDFs, primary normal human dermal fibroblasts; NHEKs, primary normal human epidermal keratinocytes; PEG, poly(ethylene glycol); SCC, squamous cell carcinoma; SK-MEL-28, skin melanoma cell line; SMCs, smooth muscle cells; T-47D, human ductal breast epithelial tumor cell line.

### 3.1. Vascular Tissues

**Figure 2:**
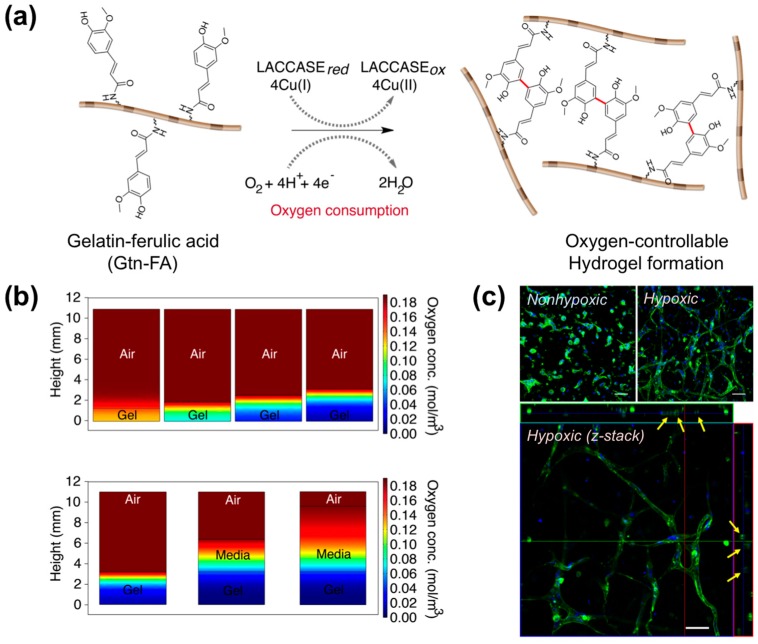
Oxygen-controllable hydrogel matrices. (**a**) A schematic illustration of hydrogel formation with oxygen consumption in the laccase-mediated crosslinking reaction; (**b**) a computer simulation of oxygen gradients throughout the hydrogel matrices, suggesting our hydrogel matrices provide an oxygen-controllable microenvironment; (**c**) confocal microscopic images of ECFCs cultured within the different oxygen levels (hypoxic *vs.* non-hypoxic); confocal z-stacks and orthogonal sections show lumen formation (indicated by arrows) within the vascular networks (phalloidin in green; nuclei in blue). Scale bars, 50 mm. From Park *et al.* [22]. Copyright 2014 with permission from Nature Publishing Group.

Newly-formed vasculature and neovascularization are essential for transplanting 3D engineered tissues with a dense cell population that may induce a necrotic core due to a lack of nutrients and oxygen supply [40,41]. Recently, many researchers have focused on developing tissue-engineered vasculature using bio-inspired hydrogel materials and various cell sources for tissue transplantation. Interestingly, many approaches have been inspired by early vascular developmental processes in embryonic microenvironments that present numerous physico-chemical and biological parameters, including cell-to-cell/matrix interactions, various soluble factors, cell adhesion molecules, varying ECM composition and remodeling and oxygen tension (reviewed by Park *et al.*) [42]. Specifically, it is well known that oxygen tension is a key parameter to regulate embryonic vascular development. The partial pressure of oxygen in the embryonic microenvironment ranges from 2% to 9%, which is defined as hypoxia [43]. Growing evidence demonstrates that oxygen deprivation is important for regulating vascular differentiation and formation during embryonic development, as well as tumor metastasis and wound healing. The cellular response to low oxygen levels is controlled by hypoxia-inducible factors (HIFs) that promote vascular developmental processes, including angiogenesis and vasculogenesis [44,45,46]. Thus, many researches have endeavored to create engineered matrices that accurately mimic the oxygen tension *in vivo*. Recently, Park and Gerecht developed oxygen-controllable hydrogel materials that provide 3D artificial hypoxic microenvironments for stimulating vascular morphogenesis of endothelial progenitor cells (EPCs) through HIF-mediated pathway activation [22]. They designed ferulic acid (FA)-conjugated gelatin (Gtn-FA) polymers that formed a 3D hydrogel network with oxygen consumption in the laccase-mediated crosslinking reaction (Figure 2a). In this reaction, the laccase catalyzes the crosslinking of each FA group with an oxygen-consuming reaction. Notably, we found that oxygen levels and gradients throughout the matrices could be easily controlled by varying numerous parameters, including polymer and laccase concentrations, the degree of ferulic acid substitution and hydrogel thickness. Interestingly, we found that thicker hydrogels (>2.5 mm) induced hypoxic microenvironments when the culture media were placed, suggesting a potential for *in vitro* applications of hydrogels (Figure 2b). Using the advanced artificial matrices, they created 3D artificial vasculature using endothelial colony-forming cells (ECFCs), a subtype of EPCs. ECFCs encapsulated within the hypoxic microenvironments exhibited extensive vascular network formation through HIF pathway activation compared to cells within non-hypoxic matrices (Figure 2c). The results suggested that these engineered vasculatures may be useful in treating vascular disorders, as well as studying vascular cell biology.

In addition to tissue regeneration, engineered vasculature is extensively utilized to study basic vascular cell biology. Chwalek *et al.* established an *in vitro* angiogenesis model to study the regulation of heterocellular communication within 3D vascular tissue models [34]. The 3D matrices were prepared using PEG-derived hydrogel materials formed though click chemistry. The PEG hydrogels were tailored with MMP-sensitive and cell adhesion sites to support 3D cell growth and matrix remodeling within the artificial matrices. Heparin molecules were also included in the matrices to enhance growth factors (GFs) binding affinity via electrical interaction between heparin and GFs. They encapsulated endothelial cells (ECs) and/or mural cells to study the effect of matrix stiffness, GFs and heterotypic cell-to-cell interactions on angiogenic events. They found that soft matrices (200 Pa) encapsulating vascular endothelial growth factor (VEGF, 5 µg/mL) stimulated increased EC vascular morphogenesis compared to stiff matrices (850 Pa) at the same conditions. They also investigated the effect of combinations of GFs (e.g., VEGFs, basic fibroblast growth factor (bFGFs), stromal-derived growth factor-1α (SDF-1α)) on the tubulogenesis of ECs and found enhanced vascular network formation on culture with a cocktail of all GFs (5 µg/mL each), as compared to that for other groups. Moreover, they performed multi-cell co-cultures, including those of ECs and mural cells that support vascular remodeling and maturation (e.g., mesenchymal stem cells (MSCs), smooth muscle cells (SMCs), human dermal fibroblasts (HDFs), 10T1/2 cells), within the synthetic matrices to mimic *in vivo* vascular microenvironments. Interestingly, the co-culture systems of ECs and MSCs exhibited more stable and mature vasculatures compared to other mural cells (e.g., SMCs, HDFs and 10T1/2 cells). With the advanced *in vitro* angiogenesis models, they also demonstrated that a 3D co-culture of ECs and MSCs within the softer matrices encapsulating GF cocktails facilitated mature vascular formation. These results suggest that these engineered vascular tissue models have a promising potential as a platform for a better understanding of vascular cell biology in healthy and disease-specific angiogenesis models.

Chen and his colleagues utilized an innovative approach to rapidly construct perfusable engineered 3D vascular tissues for the study of basic vascular cell biology and tissue regeneration [47]. They created patterned and cylindrical vascular constructs encapsulating live cells using biocompatible sacrificial templates, such as carbohydrate glass. The cylindrical surfaces were lined with ECs to mimic the inner line of the native vasculatures. In addition, they prepared the perfusable vasculatures using various cell sources and biopolymers, including agarose, alginate, PEG, fibrin and Matrigel, demonstrating that the novel technique could be adopted for a wide range of cell types, as well as polymeric hydrogels. They also utilized the co-culture systems with lined ECs and 10T1/2 cells as a stromal cell, resulting in the stromal cells surrounding the ECs and vasculature sprouting from the perfusable vessel structure toward the stromal tissues encapsulating 10T1/2 cells, which are similar biological processes to angiogenesis in the native microenvironments. Finally, they created engineered liver tissue constructs with perfusable vascular channels to retain the cellular viability and metabolic function in the thick core. Interestingly, they observed a higher cell survival in the 3D matrices with perfusable vasculature compared to without vascular channels, demonstrating that the unique approach allows enhanced cell viability and sustains the cell function even within the densely-populated tissue constructs. Taken together, these results suggested that the perfusable vascular tissue models created by the rapid casting technique have a promising potential as a flexible and dynamic platform for the study of vascular cell biology, as well as tissue regeneration.

### 3.2. Skin Tissues

The 3D engineered skin tissue models have been implicated as a promising platform for tissue transplantation and for toxicity tests in the cosmetic industry as an alternative to animal models. Various approaches have been developed to create engineered skin tissues, including models of reconstructed human epidermis (RHE), full thickness skin model, and tissue-engineered skin models (reviewed by Mathes *et al.*) [48]. In recent years, many researches have endeavored to create tissue-engineered skin models using polymeric hydrogel matrices that accurately mimic the native skin tissues. To create 3D skin tissue constructs, various cell sources have been assessed, including keratinocytes, human umbilical vein endothelial cells (HUVECs) and fibroblasts. [48]. More recently, stem cells (e.g., adult stem cells and pluripotent stem cells) have been widely used as a cell source to create tissue constructs, rather than other cell sources, due to their ability to differentiate into vascular lineages. Various engineered skin tissues are fabricated using collagen, which is abundant in skin ECMs, which can be used in the evaluation of drug efficacy, toxicity, as well as drug screening for healthy and disease skin tissues. For example, Commandeur *et al.* established 3D *in vitro* models of normal skin and human cutaneous squamous cell carcinoma (SCC) to study the molecular mechanisms of carcinoma progression through either activation or inhibition of the epidermal growth factor receptor (EGFR) [32]. The engineered models were fabricated by encapsulating multiple cell lines (e.g., primary normal human epidermal keratinocytes (NHEKs) and primary normal human dermal fibroblasts (NHDFs), SCC-12B and SCC-13 cell lines) into collagen hydrogels. To assess the effect of EGFR activation and inhibition on SCC progression, they cultured the tissue models with different EGF concentrations (5, 20 and 50 ng/mL) with or without erlotinib (10 µM) as an anti-cancer drug. Interestingly, EGF-induced EGFR activation in healthy and tumorous skin facilitated severe epidermis disorganization and invasion at higher concentrations (>20 ng/mL), suggesting that EGF is important in regulating epidermis proliferation within healthy and diseased skin tissues. They also found that the treatment of erlotinib on both healthy and SCC tissues induces the reduction of epidermal thickness, suggesting that the drug inhibited epidermal cell proliferation. Specifically, treatment of healthy skin with erlotinib induced a severe decrease in epidermal thickness as a known side effect of the drugs in patients. These results demonstrate that 3D healthy and malignant skin models can be used to study the effect of various skin cancer drugs, as well as skin cancer biology.

Tissue-engineered skin models have been used as skin grafts for treating skin defects caused by burns or chronic wounds. While engineered dermo-epidermal skin substitutes (DESS) are used instead of autologous transplantation, the initial vascularization after transplantation still needs improvement to ensure successful tissue transplantation [33]. One solution is to generate prevascularized skin grafts. Klar *et al.* developed tissue-engineered dermo-epidermal skin grafts prevascularized using collagen hydrogels with adipose-derived stem cells (ADSCs) [49]. The engineered vasculature was fabricated by encapsulating ADSCs into the hydrogel matrices and evaluated for *in vivo* performance, showing tissue homeostasis and sustained epidermal coverage *in vivo*. This result suggests that the advanced skin graft can be used in burn treatment, plastic surgery and other chronic diseases in dermatology.

### 3.3. Tumor Models

An emerging trend in cancer research is to develop engineered 3D tumor models that accurately mimic the native tumor microenvironments. Growing evidence demonstrates that tumor microenvironments are important in cancer progression and metastasis through numerous physical, chemical and biological parameters, including matric stiffness, pH, oxygen gradients, matric remodeling, matrix topography and cellular communications between cell-to-cell/matrix, as well as stromal cell signaling. Traditional 2D culture and animal models are limited in their ability to recapitulate *in vivo* tumor microenvironments due to the lack of spatiotemporal complexity and interspecies-dependent discrepancies, resulting in poor clinical outcomes [13]. Thus, various 3D tumor models have been developed using biomimetic hydrogels to study basic cancer biology and screening of newly-developed drugs or carriers for better clinical outcomes (reviewed by Song *et al.*) [50].

Pedron *et al.* reported a 3D-engineered brain tumor model using photo-crosslinkable HA and gelatin hydrogels. Using the bioinspired hydrogels combined with microfluidic devices, they fabricated the artificial tumor microenvironments with gradation of brain tumor cells (glioblastoma multiforme, GBM) and matric contents (e.g., HA and gelatin), which can precisely mimic the native tumor environments *in vivo* [35]. Using the advanced brain tumor models, they found that the gradation in cell density, matrix composition and structural architecture affect spatially-selective changes in the GBM malignant phenotype, showing a promising potential for cancer research. Recently, Shen *et al.* established a tumor-angiogenesis model using HA-based hydrogels formed via the Michael-type addition reaction [36]. To generate co-culture models, human fibrosarcomas (HT1080) were encapsulated within the HA hydrogel matrices, and ECFCs were cultured on top of the hydrogels. They investigated the effect of matrix stiffness and oxygen tension on vascular cell invasion, demonstrating that a soft matrix under atmospheric conditions had high angiogenic induction, while hypoxic environments upregulated angiogenic responses in stiffer hydrogels. These results suggest that tumor-angiogenesis models can be used to study the basic tumor biology.

More recently, Stephanie *et al.* developed a multi-tunable engineered tumor models to study breast cancer cell invasion [31]. They designed HA-based hydrogel matrices decorated with the MMP-sensitive and cell adhesion sites to support cancer cell growth within the 3D matrices, which can be crosslinked via click-reaction. The engineered breast cancer models were fabricated by culturing either poorly-invasive breast cancer cells (human breast adenocarcinoma cell line (MCF-7), human ductal breast epithelial tumor cell line (T-47D), skin melanoma cell line (SK-MEL-28) or an invasive cell line (human breast adenocarcinoma cell line (MDA-MB-231)) on the HA-based semi-synthetic matrices with different crosslinking degrees and cell adhesion ligand densities to investigate the effect of matrix stiffness and the ligand density on the breast cancer cell invasion. They first found that the invasive MDA-MB-231 cells readily penetrated into the hydrogels, while the other cell lines remained on the surfaces. They also noticed that softer matrix exhibited longer invasion distance (758 ± 78 μm) compared to stiff matrices (85 ± 62 μm), suggesting that matrix stiffness is critical to cancer cell invasion. They also studied the effect of cell adhesion ligand density on the cancer cell invasion and proliferation. Interestingly, they noticed that RGD density did not affect cancer cell invasion, while the matrices tailored with a high RGD concentration facilitated cancer cell proliferation. Finally, they examined the MMP inhibition studies using an MMP inhibitor (e.g., GM6001) to demonstrate the MMP-mediated cancer cell invasion, resulting in that cancer cell invasion occurred through MMP-mediated matrix degradation, but the cell proliferation was not affected. These results suggested that the HA-based engineered tumor models could be utilized as an emerging platform to understand the role of physico-chemical properties in the tumor microenvironments on cancer cell invasion.

More recently, Laura *et al.* also developed bioengineered tumor models to study tumor angiogenesis and drug resistance [37]. They created the engineered matrices using star-shaped PEG tailored with the MMP-sensitive sites and cell adhesion ligand, as well as heparin derivatives that can entrap the GFs within the artificial microenvironments through the specific interaction between heparin and GFs. To create vascularized tumor models, they incorporated various GFs (e.g., bFGF, VEGFs and SDF-1) to support vascular cell growth and differentiation within the matrices. Using the semi-synthetic matrices, they utilized tri-culture systems with ECs and MSCs to create capillary structures and either breast cancer cells (e.g., MDA-MB-231 and MCF-7) or prostate cancer cells (e.g., LNCaP and PC3) to generate *in vitro* tumor formation for studying tumor angiogenesis and investigating drug resistance. Notably, they found that tri-culture systems (ECs/MSCs and each cancer cell) showed enhanced drug resistance toward Epirubicin and Paclitaxel, which are FDA-approved anti-cancer drugs, compared to traditional 2D and 3D single tumor models. These results suggested that the multi-cellular engineered tumor models might provide an innovative platform to study basic cancer biology and to identify the target therapeutic drugs with their effective dose for better clinical outcomes in breast and prostate cancer treatment.

### 3.4. Other Tissue Models

Polymeric hydrogels have also been widely utilized to create other tissue models, including hepatic tissue models and pancreatic islet models. Bhatia and her colleagues developed engineered hepatic tissues for the study of basic cell biology and tissue regeneration [38]. They created artificial microenvironments using diarylated PEG with MMP-sensitive and cell adhesion sites, which can form hydrogels via a photo-crosslinking reaction. To demonstrate the effect of hepatocyte concentrations on the biological function of the *in vitro* hepatic tissue, they prepared the engineered tissue constructs with different cell concentrations (eight million per mL *vs.* four million per mL), resulting in that the higher cell concentration showed a higher level of albumin and urea synthesis, which are critical functions of liver tissues. They also transplanted the artificial hepatic tissues into the back of mice, demonstrating the *in vivo* function and stability of the engineered tissues up to Day 14. The results suggested that the engineered liver tissue could be applied to the study of basic liver biology and tissue regenerative medicine.

Various 3D pancreatic models have been developed to recapitulate the cellular microenvironment of native islet in pancreases *in vivo* (reviewed by Gao *et al.* [51]). The engineered islet tissue constructs have been widely utilized as a platform for drug screening and islet tissue engineering. Bryant and her colleagues developed engineered pancreatic islets using PEG-based hydrogels encapsulating human embryonic stem cell-derived pancreatic precursor cell aggregates through a photo-crosslinking reaction [39]. Using the platform, they investigated the effect of collagen type I on the islet aggregation formation and their biological function. They found that encapsulating collagen type I allowed the islet to form large aggregates (diameter of 85 μm at Day 14; diameter of 195 μm) compared to traditional suspension culture (diameter of 83 μm at Day 14; diameter of 100 μm). Interestingly, they also found that the islet aggregates cultured within the engineered matrix incorporating collagen showed higher cell viability after Day 28 than those cultured without collagen. These results demonstrated that collagen type I plays a pivotal role in islet aggregate formation and their viability within the pancreatic islet microenvironment. These 3D pancreatic islet tissue models can be applied as a platform into drug screening and tissue transplantation for the treatment of pancreatic disease.

## 4. Conclusions and Future Directions

Numerous engineered tissue models have been explored as emerging platforms to support extensive research, ranging from tissue regeneration to the study of basic cell biology. While polymeric matrices are extensively used to recapitulate *in vivo* cellular microenvironments, it is still required to better mimic complex and convoluted native tissues *in vivo*. In recent years, emerging macro-/nano-fabrication techniques have been developed to better mimic native cellular microenvironments, including microfluidic devices and 3D printing techniques [52,53]. These innovative approaches allow the creation of spatially-controlled 3D culture environments and heterogeneous cell-laden tissue constructs [54]. Thus, the advanced tissue models may provide unique opportunities for successful tissue regeneration, drug discovery and a better understanding of biological mechanisms in healthy and diseased tissues.

## Abbreviations

The following abbreviations are used in this manuscript:

2D: two-dimensional
3D: three-dimensional
ECMs: extracellular matrices
HA: hyaluronic acid
PVA: poly(vinyl alcohol)
PNIPAAm: poly(*N*-isopropylacrylamide)
PEO–PPO–PEO: poly(ethylene oxide)–poly(propylene oxide)–poly(ethylene oxide)
MMP: matrix metalloproteinase
GAG: glycosaminoglycan
HIFs: hypoxia-inducible factors
FA: ferulic acid
Gtn: gelatin
GFs: growth factors
VEFG: vascular endothelial growth factor
bFGF: basic fibroblast growth factor
SDF-1α: stromal-derived growth factor-1α
ADSCs: adipose-derived stem cells
ECs: endothelial cells
EPCs: endothelial progenitor cells
ECFCs: endothelial colony-forming cells
EGFR: epidermal growth factor receptor
GBM: glioblastoma multiforme
HDFs: human dermal fibroblasts
SK-MEL-28: skin melanoma cell line
HT1080: human fibrosarcomas
MCF-7: human breast adenocarcinoma cell line
MDA-MB-23: human breast adenocarcinoma cell line
MSCs: mesenchymal stem cells
NHDFs: primary normal human dermal fibroblasts
NHEKs: primary normal human epidermal keratinocytes
PEG: poly(ethylene glycol)
SCC: squamous cell carcinoma
SMCs: smooth muscle cells
T-47D: human ductal breast epithelial tumor cell line

## References

[1] Angelova N., Hunkeler D. (1999). Rationalizing the design of polymeric biomaterials. Trends Biotechnol..

[2] Kohane D.S., Langer R. (2008). Polymeric biomaterials in tissue engineering. Pediatr. Res..

[3] Lutolf M., Hubbell J. (2005). Synthetic biomaterials as instructive extracellular microenvironments for morphogenesis in tissue engineering. Nat. Biotechnol..

[4] Baudis S., Nehl F., Ligon S.C., Nigisch A., Bergmeister H., Bernhard D., Stampfl J., Liska R. (2011). Elastomeric degradable biomaterials by photopolymerization-based CAD–CAM for vascular tissue engineering. Biomed. Mater..

[5] Burdick J.A., Prestwich G.D. (2011). Hyaluronic acid hydrogels for biomedical applications. Adv. Mater..

[6] Kamata H., Li X., Chung U.I., Sakai T. (2015). Design of hydrogels for biomedical applications. Adv. Healthc. Mater..

[7] Kashyap N., Kumar N., Kumar M.N. (2005). Hydrogels for pharmaceutical and biomedical applications. Crit. Rev. Ther. Drug Carr. Syst..

[8] Seliktar D. (2012). Designing cell-compatible hydrogels for biomedical applications. Science.

[9] Spizzirri U.G., Curcio M., Cirillo G., Spataro T., Vittorio O., Picci N., Hampel S., Iemma F., Nicoletta F.P. (2015). Recent advances in the synthesis and biomedical applications of nanocomposite hydrogels. Pharmaceutics.

[10] Tomatsu I., Peng K., Kros A. (2011). Photoresponsive hydrogels for biomedical applications. Adv. Drug Deliv. Rev..

[11] Hay E.D. (2011). Cell Biology of Extracellular Matrix.

[12] Tibbitt M.W., Anseth K.S. (2009). Hydrogels as extracellular matrix mimics for 3D cell culture. Biotechnol. Bioeng..

[13] Francia G., Kerbel R.S. (2010). Raising the bar for cancer therapy models. Nat Biotechnol..

[14] DeForest C.A., Anseth K.S. (2012). Advances in bioactive hydrogels to probe and direct cell fate. Annu. Rev. Chem. Biomol. Eng..

[15] Cai L., Dewi R.E., Heilshorn S.C. (2015). Injectable hydrogels with *in situ* double network formation enhance retention of transplanted stem cells. Adv. Funct. Mater..

[16] Park K.M., Son J.Y., Choi J.H., Kim I.G., Lee Y., Lee J.Y., Park K.D. (2014). Macro/Nano-gel composite as an injectable and bioactive bulking material for the treatment of urinary incontinence. Biomacromolecules.

[17] Highley C.B., Rodell C.B., Burdick J.A. (2015). Direct 3D printing of shear-thinning hydrogels into self-healing hydrogels. Adv. Mater..

[18] Hoffman A.S. (2002). Hydrogels for biomedical applications. Adv. Drug Deliv. Rev..

[19] Lu H.D., Charati M.B., Kim I.L., Burdick J.A. (2012). Injectable shear-thinning hydrogels engineered with a self-assembling Dock-and-Lock mechanism. Biomaterials.

[20] Sivashanmugam A., Arunkumar R., Priya M.V., Nair S.V., Jayakumar R. (2015). An overview of injectable polymeric hydrogels for tissue engineering. Eur. Polym. J..

[21] Kloxin A.M., Benton J.A., Anseth K.S. (2010). *In situ* elasticity modulation with dynamic substrates to direct cell phenotype. Biomaterials.

[22] Park K.M., Gerecht S. (2014). Hypoxia-inducible hydrogels. Nat. commun..

[23] Deforest C.A., Sims E.A., Anseth K.S. (2010). Peptide-functionalized click hydrogels with independently tunable mechanics and chemical functionality for 3D cell culture. Chem. Mater. Publ. Am. Chem. Soc..

[24] Schultz K.M., Kyburz K.A., Anseth K.S. (2015). Measuring dynamic cell-material interactions and remodeling during 3D human mesenchymal stem cell migration in hydrogels. Proc. Natl. Acad.Sci. USA.

[25] Lee K.Y., Mooney D.J. (2001). Hydrogels for tissue engineering. Chem. Rev..

[26] Ben-David D., Srouji S., Shapira-Schweitzer K., Kossover O., Ivanir E., Kuhn G., Müller R., Seliktar D., Livne E. (2013). Low dose BMP-2 treatment for bone repair using a PEGylated fibrinogen hydrogel matrix. Biomaterials.

[27] Blatchley M., Park K.M., Gerecht S. (2015). Designer hydrogels for precision control of oxygen tension and mechanical properties. J. Mater. Chem. B.

[28] Rice J.J., Martino M.M., de Laporte L., Tortelli F., Briquez P.S., Hubbell J.A. (2013). Engineering the regenerative microenvironment with biomaterials. Adv. Healthc. Mater..

[29] Cho H.-J., Yoon H.Y., Koo H., Ko S.-H., Shim J.-S., Lee J.-H., Kim K., Kwon I.C., Kim D.-D. (2011). Self-assembled nanoparticles based on hyaluronic acid-ceramide (HA-CE) and Pluronic^®^ for tumor-targeted delivery of docetaxel. Biomaterials.

[30] Yu M., Jambhrunkar S., Thorn P., Chen J., Gu W., Yu C. (2013). Hyaluronic acid modified mesoporous silica nanoparticles for targeted drug delivery to CD44-overexpressing cancer cells. Nanoscale.

[31] Fisher S.A., Anandakumaran P.N., Owen S.C., Shoichet M.S. (2015). Tuning the microenvironment: Click-crosslinked hyaluronic acid-based hydrogels provide a platform for studying breast cancer cell invasion. Adv. Funct. Mater..

[32] Commandeur S., van Drongelen V., de Gruijl F.R., El Ghalbzouri A. (2012). Epidermal growth factor receptor activation and inhibition in 3D *in vitro* models of normal skin and human cutaneous squamous cell carcinoma. Cancer Sci..

[33] Klar A.S., Bottcher-Haberzeth S., Biedermann T., Schiestl C., Reichmann E., Meuli M. (2014). Analysis of blood and lymph vascularization patterns in tissue-engineered human dermo-epidermal skin analogs of different pigmentation. Pediatr. Surg. Int..

[34] Chwalek K., Tsurkan M.V., Freudenberg U., Werner C. (2014). Glycosaminoglycan-based hydrogels to modulate heterocellular communication in *in vitro* angiogenesis models. Sci. Rep..

[35] Pedron S., Becka E., Harley B.A. (2015). Spatially gradated hydrogel platform as a 3D engineered tumor microenvironment. Adv. Mater..

[36] Shen Y.-I., Abaci H.E., Krupski Y., Weng L.-C., Burdick J.A., Gerecht S. (2014). Hyaluronic acid hydrogel stiffness and oxygen tension affect cancer cell fate and endothelial sprouting. Biomater. Sci..

[37] Bray L.J., Binner M., Holzheu A., Friedrichs J., Freudenberg U., Hutmacher D.W., Werner C. (2015). Multi-parametric hydrogels support 3D *in vitro* bioengineered microenvironment models of tumour angiogenesis. Biomaterials.

[38] Stevens K.R., Miller J.S., Blakely B.L., Chen C.S., Bhatia S.N. (2015). Degradable hydrogels derived from PEG-diacrylamide for hepatic tissue engineering. J. Biomed.Mater. Res. A.

[39] Amer L.D., Holtzinger A., Keller G., Mahoney M.J., Bryant S.J. (2015). Enzymatically degradable poly(ethylene glycol) hydrogels for the 3D culture and release of human embryonic stem cell derived pancreatic precursor cell aggregates. Acta Biomater..

[40] Jain R.K., Au P., Tam J., Duda D.G., Fukumura D. (2005). Engineering vascularized tissue. Nat. Biotechnol..

[41] Rouwkema J., Rivron N.C., van Blitterswijk C.A. (2008). Vascularization in tissue engineering. Trends Biotechnol..

[42] Park K.M., Gerecht S. (2014). Harnessing developmental processes for vascular engineering and regeneration. Development.

[43] Simon M.C., Keith B. (2008). The role of oxygen availability in embryonic development and stem cell function. Nat. Rev. Mol. Cell Biol..

[44] De Bock K., Mazzone M., Carmeliet P. (2011). Antiangiogenic therapy, hypoxia, and metastasis: Risky liaisons, or not?. Nat. Rev.Clin. oncol..

[45] Keith B., Johnson R.S., Simon M.C. (2012). HIF1α and HIF2α: Sibling rivalry in hypoxic tumour growth and progression. Nat. Rev. Cancer.

[46] Majmundar A.J., Wong W.J., Simon M.C. (2010). Hypoxia-inducible factors and the response to hypoxic stress. Mol. Cell.

[47] Miller J.S., Stevens K.R., Yang M.T., Baker B.M., Nguyen D.-H.T., Cohen D.M., Toro E., Chen A.A., Galie P.A., Yu X. (2012). Rapid casting of patterned vascular networks for perfusable engineered three-dimensional tissues. Nat. Mater..

[48] Mathes S.H., Ruffner H., Graf-Hausner U. (2014). The use of skin models in drug development. Adv. Drug Deliv. Rev..

[49] Klar A.S., Guven S., Biedermann T., Luginbuhl J., Bottcher-Haberzeth S., Meuli-Simmen C., Meuli M., Martin I., Scherberich A., Reichmann E. (2014). Tissue-engineered dermo-epidermal skin grafts prevascularized with adipose-derived cells. Biomaterials.

[50] Song H.H., Park K.M., Gerecht S. (2014). Hydrogels to model 3D *in vitro* microenvironment of tumor vascularization. Adv. Drug Deliv. Rev..

[51] Gao B., Wang L., Han S., Pingguan-Murphy B., Zhang X., Xu F. (2015). Engineering of microscale three-dimensional pancreatic islet models *in vitro* and their biomedical applications. Crit. Rev. Biotechnol..

[52] Murphy S.V., Atala A. (2014). 3D bioprinting of tissues and organs. Nat. Biotechnol..

[53] Van Duinen V., Trietsch S.J., Joore J., Vulto P., Hankemeier T. (2015). Microfluidic 3D cell culture: from tools to tissue models. Curr. opin. Biotechnol..

[54] Kolesky D.B., Truby R.L., Gladman A.S., Busbee T.A., Homan K.A., Lewis J.A. (2014). 3D bioprinting of vascularized, heterogeneous cell-laden tissue constructs. Adv. Mater..

